# Chemical Scaffolds Driving Modern Anticancer Drug Discovery and Radiotheranostics: Structural Determinants, Translational Opportunities and Future Perspectives

**DOI:** 10.3390/ph19081248

**Published:** 2026-08-08

**Authors:** Marta Rusek

**Affiliations:** Independent Unit of Radiopharmacy, Faculty of Pharmacy, Medical University of Lublin, 20-093 Lublin, Poland; marta.rusek@umlub.edu.pl

**Keywords:** anticancer agents, chemical scaffolds, medicinal chemistry, radiotheranostics, radiopharmaceuticals, thiosemicarbazones, molecular imaging, targeted radionuclide therapy, copper radionuclides, precision oncology

## Abstract

Cancer remains one of the leading causes of morbidity and mortality worldwide despite advances in molecular oncology and targeted therapeutics. The growing demand for precision medicine has accelerated the development of radiotheranostics, an emerging paradigm that integrates molecular imaging and targeted radionuclide therapy. In parallel, medicinal chemistry continues to generate structurally diverse small-molecule scaffolds capable of modulating key oncogenic pathways. Increasing evidence indicates that certain chemical scaffolds possess intrinsic properties that extend beyond conventional anticancer activity and support their translation into radiotheranostic applications. This review examines major scaffold classes driving contemporary anticancer drug discovery, including thiosemicarbazones, heterocyclic compounds, metal-based agents, hybrid molecules, and multifunctional platforms. Particular attention is given to the structural features governing biological activity, target selectivity, metal coordination, and radiolabeling potential. The review further highlights the mechanistic convergence between scaffold-mediated anticancer effects and radionuclide-induced cytotoxicity, emphasizing shared pathways involving DNA damage, oxidative stress, inhibition of DNA repair, and modulation of oncogenic signaling. Based on these observations, a scaffold-centered framework for radiotheranostic development is proposed, with perspectives on hybrid molecular design, copper-based theranostic systems, and artificial intelligence-assisted ligand discovery. By integrating medicinal chemistry, molecular oncology, and nuclear medicine, this review outlines structural principles that may facilitate the rational design of next-generation precision anticancer agents and radiotheranostic platforms.

## 1. Introduction

Cancer remains one of the greatest global health challenges, accounting for millions of deaths annually and imposing an increasing socioeconomic burden on healthcare systems worldwide. Despite advances in molecular oncology, early diagnosis, and targeted therapeutics, many malignancies continue to exhibit poor clinical outcomes due to tumor heterogeneity, therapeutic resistance, metastatic progression, and limited treatment selectivity. Consequently, the advancement of innovative therapeutic strategies capable of improving efficacy while minimizing systemic toxicity remains a central objective of contemporary cancer research [1,2,3].

**Figure 1:**
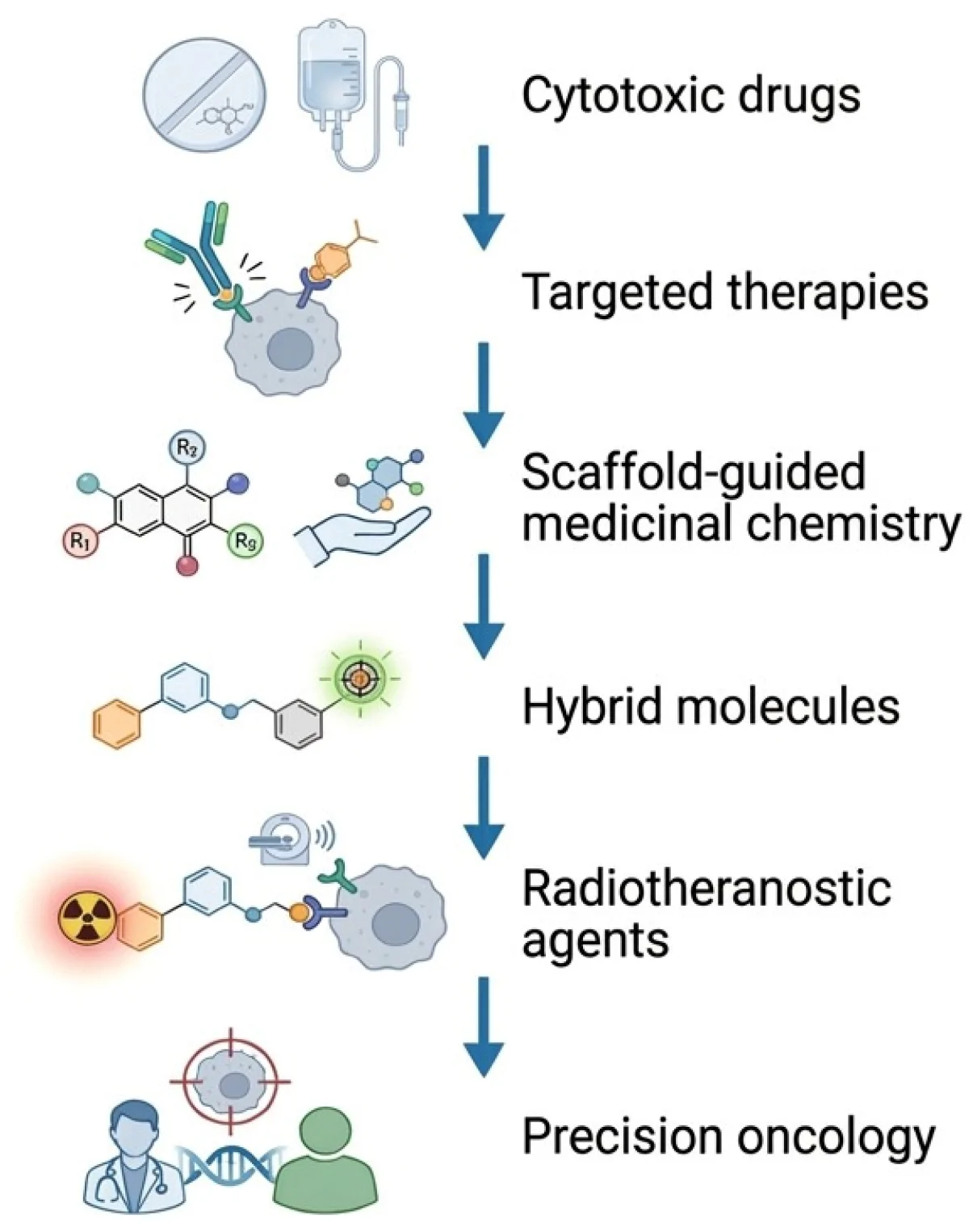
Evolution of anticancer drug discovery toward precision radiotheranostics. Advances in scaffold-based medicinal chemistry have transformed anticancer drug development from conventional cytotoxic agents to targeted therapies and integrated radiotheranostic platforms, supporting modern precision oncology. Created in Figure Labs: https://chat.figurelabs.ai/verify/FL-PUB-20260628-JV3UGQ (accessed on 28 July 2026).

Over the past two decades, the paradigm of anticancer drug discovery has evolved from non-specific cytotoxic chemotherapy toward mechanism-based precision oncology. Advances in genomics, transcriptomics, and molecular pathology have enabled the identification of actionable molecular targets, leading to the development of kinase inhibitors, DNA repair modulators, epigenetic regulators, and immune-directed therapies [1,2,4]. This evolution can be conceptually viewed as a transition from broadly cytotoxic agents toward molecularly defined therapeutic platforms (Figure 1). In this trajectory, chemical scaffolds serve as the structural link between target recognition, biological activity, and further adaptation into imaging or radionuclide-based therapeutic agents. More recently, artificial intelligence (AI)-assisted drug discovery and structure-based computational design have further accelerated the identification and optimization of molecular frameworks suitable for precision oncology [5].

Nevertheless, even highly selective targeted agents frequently encounter limitations associated with acquired resistance, insufficient tumor penetration, off-target effects, and the dynamic evolution of cancer cell populations [6]. These challenges have stimulated continued interest in the discovery of new molecular entities capable of engaging multiple oncogenic pathways while retaining favorable pharmacological properties. Within this context, chemical scaffolds represent the molecular foundation of medicinal chemistry. This molecular framework provides the structural basis upon which biological activity, target recognition, physicochemical behavior, and pharmacokinetic characteristics are built [7].

**Figure 2:**
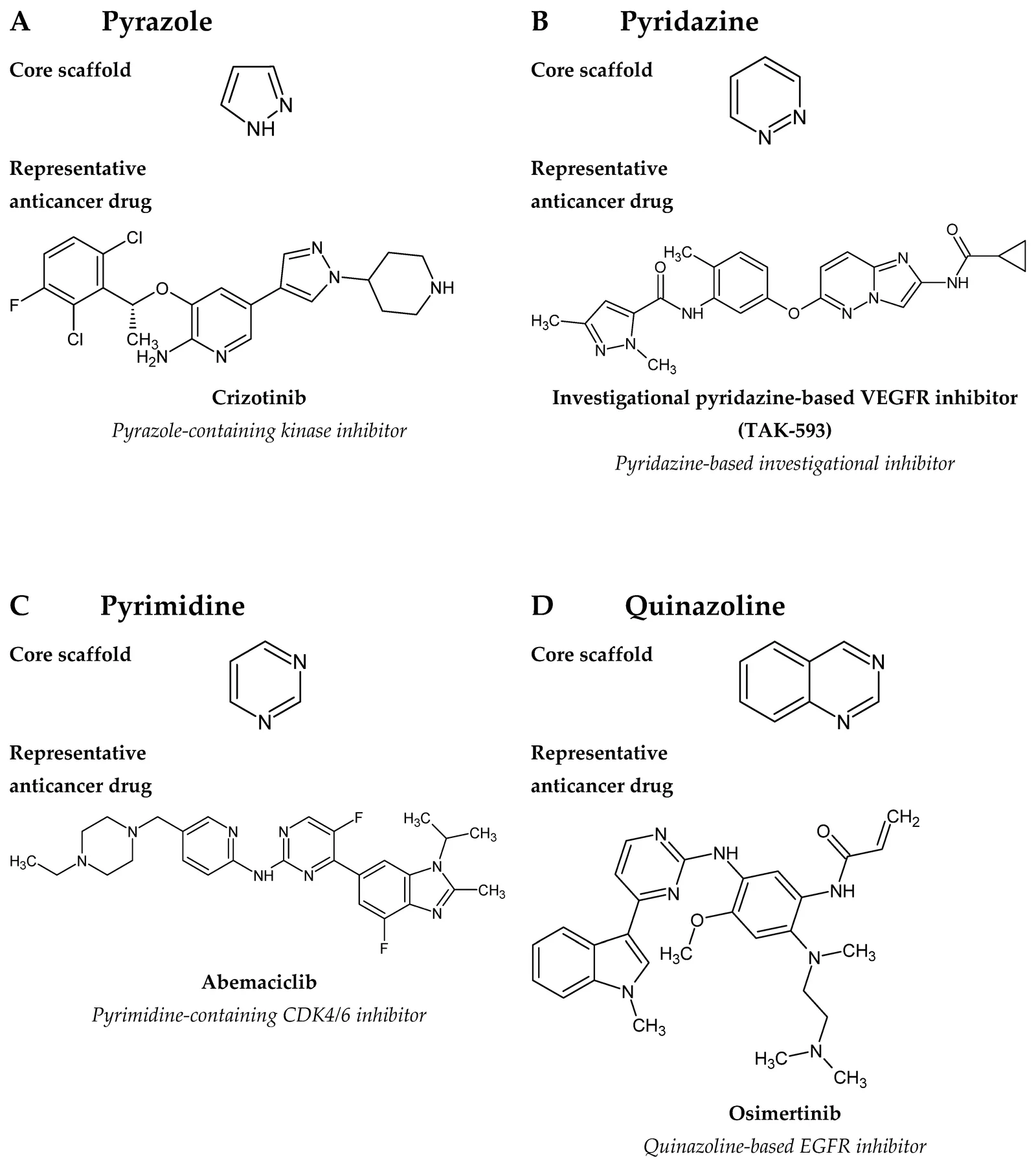
Representative privileged heterocyclic scaffolds and corresponding anticancer agents. Panels (**A**–**D**) show representative heterocyclic scaffolds (**top**) and selected anticancer agents incorporating these scaffolds (**bottom**): (**A**) pyrazole—Crizotinib, (**B**) pyridazine—TAK-593, (**C**) pyrimidine—Abemaciclib, and (**D**) quinazoline—Osimertinib. ALK—anaplastic lymphoma kinase; CDK4/6—cyclin-dependent kinase 4/6; EGFR—epidermal growth factor receptor; VEGFR—vascular endothelial growth factor receptor.

The selection of an appropriate scaffold often determines not only the potency of a compound but also its synthetic accessibility, optimization potential, and translational feasibility. Therefore, scaffold-centered approaches have become fundamental to modern anticancer drug design, enabling the rational optimization of compounds with improved efficacy, selectivity, and multifunctionality [7,8]. Several structural classes have emerged as particularly important in oncology. Heterocyclic systems such as pyrazoles, pyridazines, pyrimidines, and quinazolines constitute the core structures of numerous kinase inhibitors and targeted therapeutics [9]. Quinazoline scaffolds gave rise to epidermal growth factor receptor (EGFR) inhibitors such as Gefitinib and Osimertinib, pyrimidine derivatives underpin cyclin depend kinase 4/6 (CDK4/6) inhibitors including Abemaciclib, whereas pyrazole-containing architectures contributed to the development of kinase inhibitors such as Crizotinib. Representative core structures and selected anticancer agents derived from these heterocyclic frameworks are presented in Figure 2. The comparison shows how relatively simple privileged scaffolds can be systematically functionalized to generate structurally complex and clinically relevant targeted therapeutics.

Thiosemicarbazones have attracted growing attention because of their metal-chelating properties, ribonucleotide reductase inhibition, and redox-modulating activity [10,11]. Metal-based compounds continue to expand beyond traditional platinum drugs, incorporating copper, ruthenium, and gold complexes with diverse mechanisms of action [10,12,13,14]. Also, hybrid and multitarget-directed architectures have been developed to simultaneously modulate multiple pathways involved in tumor growth, survival, and resistance [15]. Together, these scaffold classes highlight the diversity of structural approaches currently driving anticancer drug discovery.

In parallel with advances in medicinal chemistry, nuclear medicine has undergone a profound transformation through the emergence of radiotheranostics [16]. This rapidly expanding field integrates diagnostic imaging and targeted radionuclide therapy using chemically related or identical molecular platforms [17]. Unlike conventional therapeutic approaches, radiotheranostics enables visualization of target expression, assessment of tumor heterogeneity, prediction of therapeutic response, and delivery of localized radiation to malignant tissues. The clinical success of prostate-specific membrane antigen (PSMA)-targeted agents and fibroblast activation protein inhibitor (FAPI)-based radiopharmaceuticals has demonstrated the power of this strategy and established radiotheranostics as a major pillar of precision oncology [18,19,20,21,22,23]. Additional clinically relevant radiotheranostic platforms include somatostatin receptor (SSTR)-targeting peptides used in neuroendocrine tumors, further illustrating the broad applicability of scaffold-guided radiopharmaceutical evolution [24,25,26].

The convergence of medicinal chemistry and radiopharmaceutical science has created new opportunities for scaffold-driven innovation, but not all anticancer scaffolds are equally suitable for radiotheranostic implementation. Successful radiotheranostic platforms require a combination of properties extending beyond conventional biological activity, including high target affinity, structural modularity, favorable pharmacokinetics, compatibility with radionuclide incorporation, and the ability to maintain biological function following radiolabeling [17,21,22]. Besides target affinity and pharmacokinetic performance, successful translation also depends on compatibility with appropriate radionuclides, preservation of biological activity after radiolabeling, and sufficient in vivo stability to enable high-contrast imaging or effective radionuclide delivery [24,25,27]. Certain molecular classes inherently possess these characteristics and therefore offer unique opportunities for translation into imaging and therapeutic applications. Among these, thiosemicarbazones are particularly noteworthy because their intrinsic metal-chelating capacity facilitates radiometal coordination while simultaneously providing anticancer activity through modulation of metal homeostasis and redox biology [10,11]. Likewise, heterocyclic frameworks form the basis of numerous targeted inhibitors that can be adapted into radiolabeled probes for molecular imaging [9]. Also, hybrid architectures expand these possibilities by integrating targeting motifs, therapeutic pharmacophores, and radionuclide-binding functionalities within a single molecular framework [28,29]. Such developments underscore a broader shift from viewing chemical scaffolds as pharmacological backbones toward recognizing them as multifunctional platforms capable of supporting diagnosis, therapy, and patient stratification.

Despite the growing importance of radiotheranostics, most reviews continue to discuss anticancer scaffolds and radiopharmaceuticals as independent fields. Comparatively little attention has been given to the structural features that determine scaffold suitability for radiotheranostic applications. Rather than providing a comprehensive overview of all radiopharmaceuticals currently used in oncology, this review focuses on the structural characteristics of chemical scaffolds that promote successful translation into radiotheranostic applications. Where appropriate, representative clinically established radiotheranostic platforms are discussed to place scaffold-driven medicinal chemistry into a translational and clinical context.

## 2. Scaffold-Guided Anticancer Design

The success of modern anticancer drug discovery is closely linked to the identification and optimization of chemical scaffolds capable of selectively modulating disease-relevant biological targets. While individual functional groups contribute to molecular recognition and pharmacological activity, the scaffold serves as the fundamental structural framework that governs molecular geometry, electronic properties, physicochemical characteristics, and overall biological behavior. For this reason, scaffold selection represents one of the most critical decisions during the early stages of medicinal chemistry programs and frequently determines the long-term success of lead optimization and translational advancement. Beyond providing biological activity, scaffold selection increasingly determines whether a molecule can ultimately be translated into a clinically useful radiotheranostic agent through its influence on radiolabeling compatibility, molecular recognition, in vivo stability, and compatibility with clinically relevant radionuclides. Consequently, structural features established during early medicinal chemistry optimization often determine the feasibility of subsequent radiopharmaceutical development.

**Figure 3:**
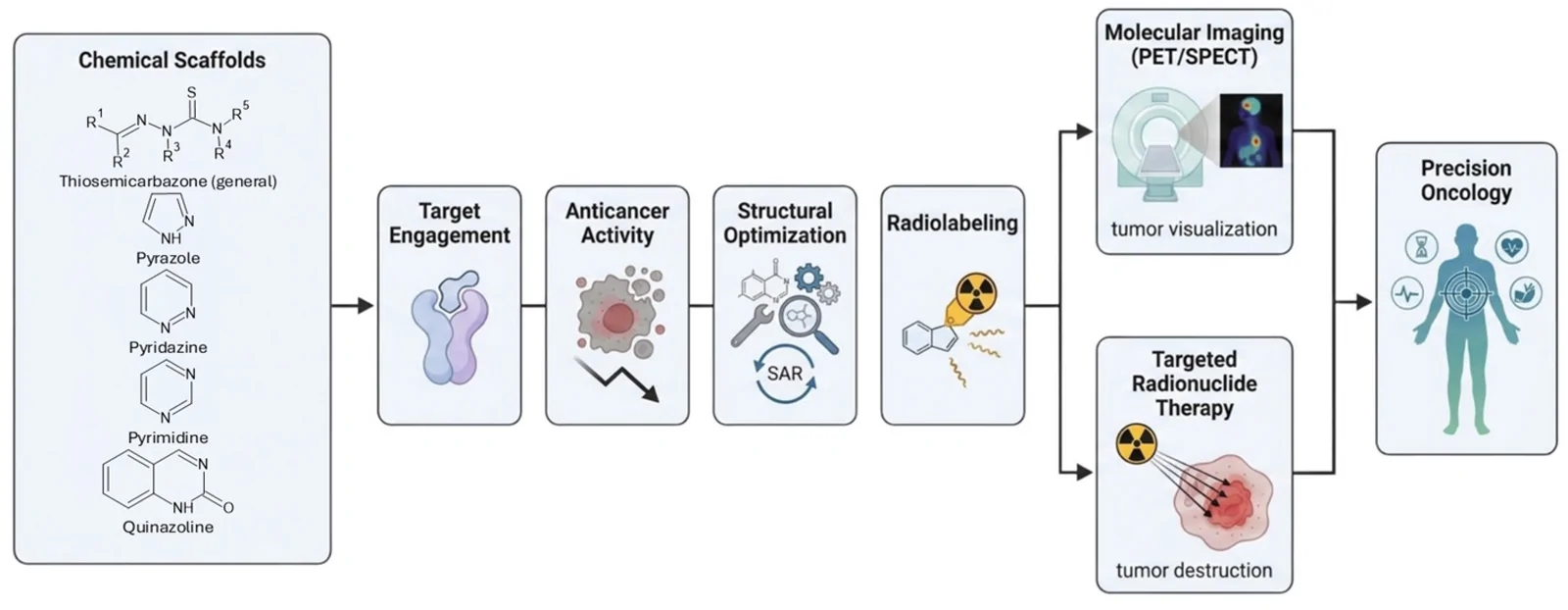
Conceptual scaffold-driven radiotheranostic development pipeline. Chemical scaffolds serve as the foundation for anticancer drug discovery and radiotheranostic development, linking target-specific therapy with molecular imaging and targeted radionuclide treatment. PET—Positron Emission Tomography; SPECT—Single-Photon Emission Computed Tomography. Created in BioRender. Rusek, M. (2026) https://BioRender.com/kaxabiy (accessed on 28 July 2026).

Historically, anticancer drug discovery relied heavily on empirical screening approaches that identified active compounds without necessarily understanding the structural principles underlying their activity. Advances in structural biology, computational chemistry, and molecular pharmacology have transformed this process, enabling the rational design of molecules based on scaffold architecture and target-specific interactions. Thus, scaffold-centered strategies have become a cornerstone of contemporary medicinal chemistry, facilitating the development of compounds with improved potency, selectivity, safety, and pharmacokinetic performance [30]. In anticancer drug discovery, quinazoline, pyrimidine, pyrazole, and indole rings are well-established examples of privileged heterocyclic scaffolds. Their planar aromatic character, hydrogen-bonding capacity, and ability to mimic nucleotide-derived interactions make them particularly suitable for kinase inhibition and other target-directed strategies [31,32,33]. The conceptual workflow linking scaffold selection with radiotheranostic progression is highlighted in Figure 3.

### 2.1. Privileged Scaffolds

A central concept in scaffold-based drug design is that of privileged scaffolds. These structural frameworks possess intrinsic characteristics that enable productive interactions with multiple classes of biological targets [30]. An effective medicinal chemistry scaffold should combine synthetic accessibility, structural robustness, favorable physicochemical properties, opportunities for systematic derivatization, and compatibility with pharmacophore optimization. In oncology, heterocyclic systems such as pyrazoles, pyridazines, quinazolines, pyrimidines, and indoles are widely recognized as privileged scaffolds because they can mimic endogenous substrates, establish hydrogen-bonding networks, and occupy enzyme binding pockets with high efficiency [7,34]. Their versatility has led to widespread application in kinase inhibitors, DNA repair modulators, epigenetic regulators, and receptor-targeted therapeutics. Examples include quinazoline-derived EGFR inhibitors, which have served as templates for radiolabeled imaging probes, and peptide-based scaffold systems such as PSMA- and somatostatin receptor-targeting ligands, illustrating how appropriate scaffold architecture enables both molecular targeting and radionuclide incorporation [35,36,37,38].

### 2.2. Structure–Activity Relationships

**Figure 4:**
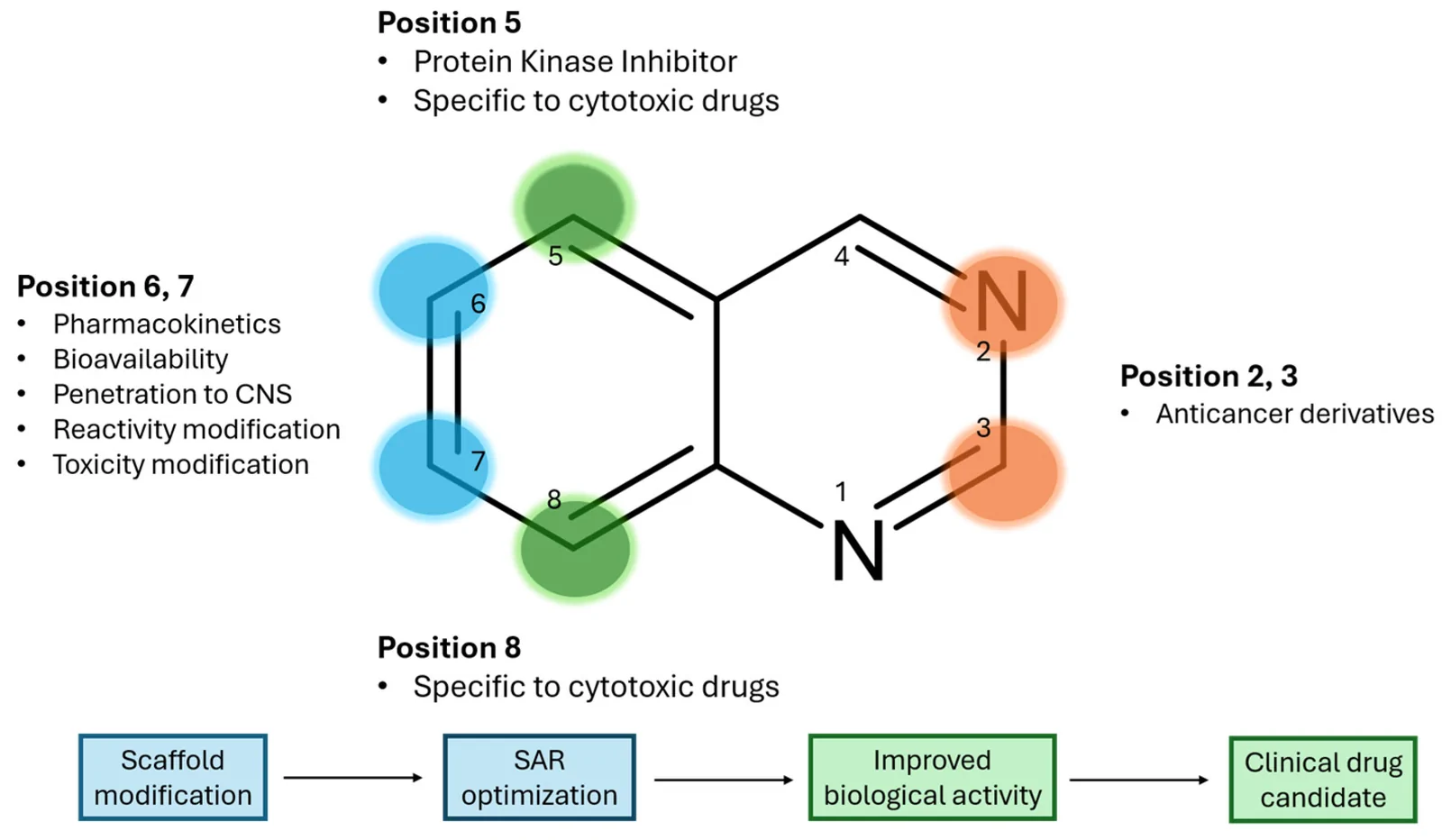
Representative examples of structure–activity relationship (SAR) optimization illustrating how systematic modification of conserved scaffolds improves target affinity, selectivity, pharmacokinetic properties, and clinical performance. The EGFR inhibitor series serves as a representative example of scaffold optimization. Based on [42].

The optimization of scaffold properties is largely guided by structure–activity relationship (SAR) analysis. Through systematic modification of substituents, ring systems, and linker regions, medicinal chemists can identify structural features responsible for biological activity and refine molecular interactions, membrane permeability, aqueous solubility, selectivity, and toxicity [39,40]. These considerations are particularly important in oncology, where therapeutic efficacy often depends on achieving sufficient tumor exposure while minimizing adverse effects in healthy tissues [41]. A representative example of SAR-guided optimization is the development of EGFR inhibitors based on the quinazoline scaffold (Figure 4) [42]. Systematic modification of substituents around the quinazoline core, particularly at positions associated with hinge binding and solvent exposure, enabled progressive improvements in potency, kinase selectivity, pharmacokinetic behavior, and activity against resistance-associated EGFR mutations. This demonstrates how relatively small structural changes within a conserved scaffold may substantially alter biological and translational performance [43,44].

### 2.3. Scaffold Hopping

One of the most influential strategies in modern medicinal chemistry is scaffold hopping, a design approach that replaces a core molecular framework with an alternative scaffold while preserving key pharmacophoric elements. Scaffold hopping is widely applied to overcome limitations associated with intellectual property constraints, poor pharmacokinetics, toxicity, or resistance mechanisms [8,45]. In oncology, this strategy has generated numerous clinically successful compounds by maintaining target engagement while improving drug-like properties [8,45]. From a radiotheranostic perspective, scaffold hopping may additionally facilitate radionuclide incorporation or enhance imaging performance without compromising biological activity. This strategy may also improve chelator positioning, optimize linker geometry, or preserve target affinity following radiolabeling, thereby enhancing the translational potential of scaffold-based radiopharmaceuticals.

Scaffold hopping has been successfully applied during the evolution of EGFR inhibitors, where structural replacement and optimization of the quinazoline core and associated substituents enabled the development of successive inhibitor generations with improved selectivity and activity against resistance-associated EGFR mutations, leading to third-generation agents such as osimertinib [8,44].

### 2.4. Molecular Hybridization

Another major trend in contemporary anticancer drug discovery is molecular hybridization. This approach combines two or more pharmacologically active motifs within a single molecular entity to create multifunctional compounds capable of simultaneously modulating distinct biological pathways [46]. Hybrid molecules have emerged as an effective strategy to address the molecular complexity of cancer by simultaneously modulating multiple interconnected signaling pathways involved in tumor progression and therapeutic resistance [29,47]. By integrating complementary pharmacophores within a unified scaffold architecture, hybrid compounds may achieve enhanced efficacy while reducing the likelihood of resistance development.

Examples of successful hybridization strategies include kinase inhibitor-histone deacetylase (HDAC) inhibitor conjugates, poly(ADP-ribose) polymerase (PARP) inhibitor-DNA-damaging agent hybrids, DNA intercalator-reactive oxygen species (ROS) generator systems, and thiosemicarbazone–heterocycle combinations [28,47]. Beyond their therapeutic advantages, hybrid architectures offer unique opportunities for radiotheranostic impact because they can simultaneously incorporate targeting ligands, therapeutic pharmacophores, and radionuclide-chelating functionalities [48,49]. Such multifunctional designs represent an important step toward the creation of integrated diagnostic and therapeutic platforms.

### 2.5. Multitarget-Directed Ligands

Closely related to molecular hybridization is the concept of multitarget-directed ligands (MTDLs). Unlike highly selective inhibitors designed to interact with a single target, MTDLs intentionally engage in multiple disease-associated pathways [50,51]. This strategy reflects an increasing recognition that complex diseases such as cancer often require network-level intervention rather than isolated target inhibition [15,29]. Therefore, the ability of certain scaffold classes to support multitarget activity is an important consideration in its selection. Thiosemicarbazones, for example, may simultaneously influence metal homeostasis, redox biology, DNA synthesis, and DNA repair, whereas heterocyclic kinase inhibitors often exhibit activity against multiple members of a signaling pathway [15].

The criteria used to evaluate scaffold quality are evolving in response to the emergence of radiotheranostics. Traditionally, medicinal chemistry optimization focused primarily on potency, selectivity, and pharmacokinetics [51]. However, scaffold suitability for radiotheranostic translation requires additional considerations, including compatibility with radiolabeling procedures, preservation of target affinity following radionuclide incorporation, in vivo stability of radiolabeled complexes, favorable biodistribution, and efficient target internalization [50,51].

Furthermore, successful clinical translation depends on the selection of radionuclides with appropriate physical half-lives, emission characteristics, radionuclide availability, and dosimetric properties that match the intended diagnostic or therapeutic application. Although these parameters extend beyond classical medicinal chemistry optimization, they should be considered during scaffold selection to allow the advancement of clinically translatable radiotheranostic agents. As radiotheranostics becomes an integral component of precision oncology, scaffold-guided medicinal chemistry will play an increasingly important role in bridging molecular design with clinically translatable imaging and targeted radionuclide therapy.

## 3. Major Anticancer Scaffolds

Chemical scaffolds constitute the structural foundation of modern anticancer drug discovery and increasingly influence the development of radiotheranostic agents. Beyond serving as molecular frameworks that support biological activity, scaffolds determine target recognition, physicochemical behavior, synthetic accessibility, and compatibility with radionuclide incorporation. While numerous scaffold classes have demonstrated anticancer potential, only a subset possesses characteristics that facilitate successful translation into radiotheranostic applications. These include structural modularity, favorable pharmacokinetics, target selectivity, metal-binding capability, and the ability to maintain biological activity following radiolabeling. This section highlights representative scaffold classes that have played significant roles in contemporary oncology and demonstrates how their structural properties contribute to both therapeutic activity and radiotheranostic potential. Rather than providing an exhaustive catalogue of anticancer agents, this section focuses on scaffold characteristics that determine successful translation into radiotheranostic applications while highlighting representative clinically established and emerging examples.

### 3.1. Thiosemicarbazones

Thiosemicarbazones (TSCs) represent one of the most versatile classes of anticancer scaffolds because they integrate potent biological activity, structural flexibility, and intrinsic transition-metal coordination within a single molecular framework. Unlike most privileged heterocyclic systems, TSCs simultaneously combine pharmacological activity with metal-binding capability, making them highly attractive for radiotheranostic applications [11,52,53,54].

**Figure 5:**
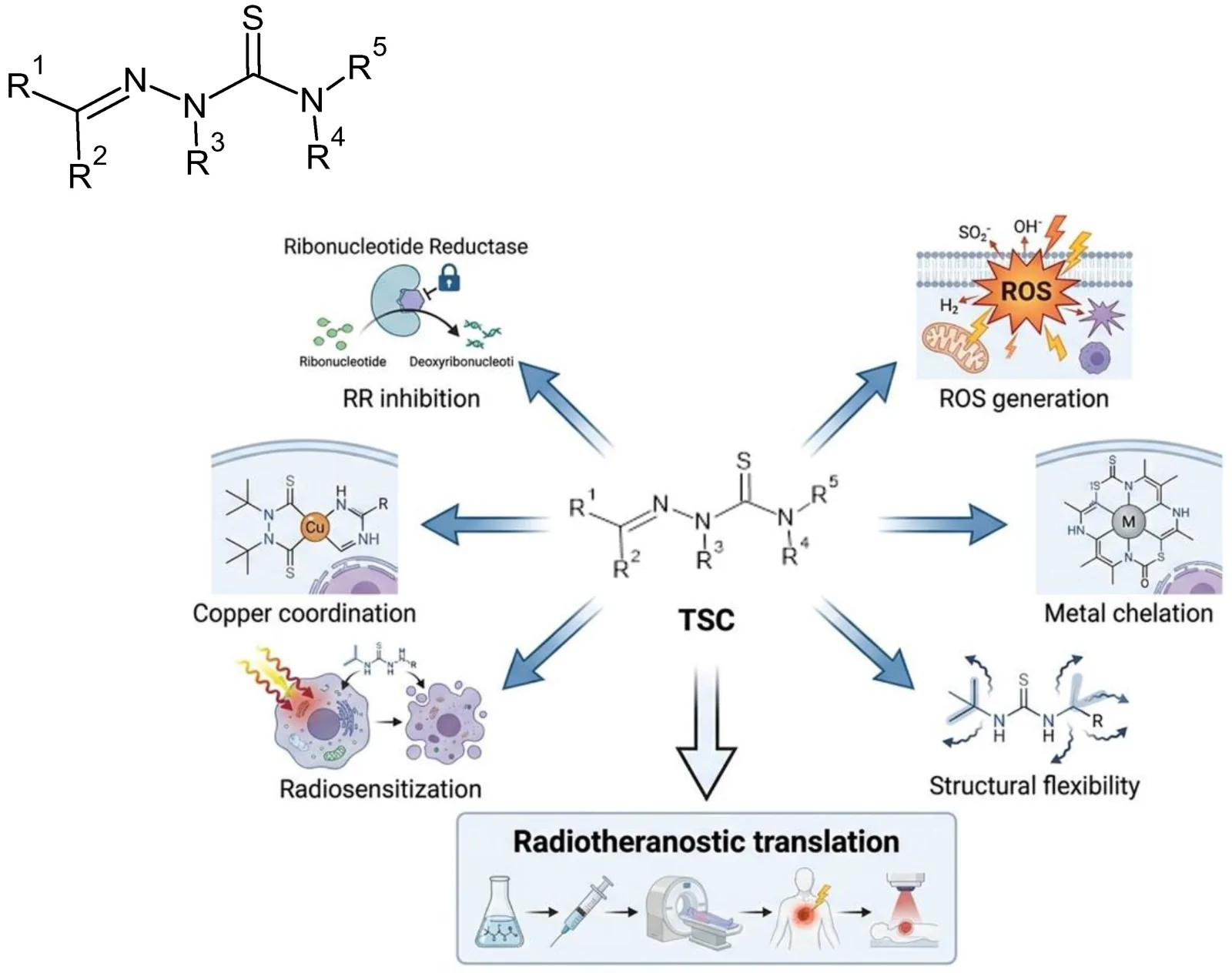
Structural architecture and multifunctional properties of thiosemicarbazones. The upper panel illustrates the general thiosemicarbazone scaffold highlighting positions available for medicinal chemistry optimization (R^1^–R^5^). The lower panel summarizes the principal biological activities supporting radiotheranostic translation, including ribonucleotide reductase inhibition, reactive oxygen species generation, metal chelation, copper coordination, radiosensitization, and structural flexibility. Created in Figure Labs: https://chat.figurelabs.ai/verify/FL-PUB-20260628-UC5IAA (accessed on 28 July 2026).

From a medicinal chemistry perspective, the thiosemicarbazone pharmacophore offers several structural advantages. The presence of multiple donor atoms enables stable coordination with biologically relevant transition metals, whereas extensive opportunities for substitution around both the aldehyde/ketone fragment and terminal nitrogen atoms allow systematic modulation of lipophilicity, electronic properties, target affinity, and pharmacokinetic behavior [55,56]. Therefore, relatively small structural modifications frequently result in substantial differences in biological activity and therapeutic selectivity (Figure 5).

One of the best-characterized mechanisms underlying the anticancer activity of TSCs is inhibition of ribonucleotide reductase (RR), the rate-limiting enzyme responsible for de novo DNA synthesis. By disrupting intracellular iron homeostasis, many TSC derivatives inhibit RR activity, impair DNA synthesis, and induce cell-cycle arrest [52,53,54]. Furthermore, metal-TSC complexes frequently promote reactive oxygen species generation, resulting in oxidative stress, mitochondrial dysfunction, and apoptosis [52,53,57]. Representative compounds such as Triapine and di-2-pyridylketone-derived TSCs have demonstrated potent activity against a wide range of tumor models and have stimulated continued interest in this scaffold class [11,58]. These biological mechanisms also provide a strong rationale for combining TSC-based compounds with targeted radionuclide therapy. Increased oxidative stress and impaired DNA repair may enhance tumor sensitivity to ionizing radiation, thereby supporting synergistic therapeutic effects. Despite these promising characteristics, several limitations should also be considered. The biological behavior of thiosemicarbazones is highly dependent on their metal coordination chemistry, lipophilicity, and intracellular redox environment. Moreover, the mechanisms responsible for Cu-ATSM retention are now recognized to be more complex than hypoxia alone and may vary between tumor types. Potential off-target accumulation, biodistribution variability, and the need for careful optimization of complex stability remain important challenges for successful clinical translation.

Another feature of TSCs is their intrinsic metal-chelating capability. TSCs naturally form stable complexes with transition metals, facilitating the development of metal-based radiopharmaceuticals [52,53]. This property is particularly relevant for copper theranostics employing the diagnostic and therapeutic radionuclides ^64Cu and ^67Cu [13,14], while the clinical and preclinical evaluation of copper(II)-diacetyl-bis(N4-methylthiosemicarbazone) (Cu-ATSM) derivatives gives the translational potential of thiosemicarbazone coordination chemistry, while also highlighting the challenges associated with tracer retention mechanisms, patient selection, and clinical implementation [59,60].

Noteworthy, the modular nature of TSC chemistry enables the incorporation of targeting ligands, heterocyclic pharmacophores, and pharmacokinetic modifiers without compromising metal-binding functionality. This combination of structural adaptability, metal coordination, and established anticancer mechanisms distinguishes TSCs from most other scaffold classes discussed in this review and positions them as highly attractive molecular platforms for future radiotheranostics.

### 3.2. Heterocyclic Scaffolds

Heterocyclic compounds constitute one of the most important classes of small molecules in medicinal chemistry and are extensively represented among clinically approved anticancer drugs [9,36]. Their structural diversity, synthetic accessibility, and ability to establish multiple non-covalent interactions with biological macromolecules make them particularly attractive scaffolds for rational drug design [7,36]. Nitrogen-containing heterocycles, including pyrazoles, pyridazines, quinazolines, and pyrimidines, are especially valuable because they readily participate in hydrogen bonding, π–π stacking, and electrostatic interactions within enzyme active sites and receptor binding pockets [7,44]. Consequently, these scaffold families have become fundamental structural motifs in kinase inhibitors, DNA damage response modulators, and other targeted anticancer agents [44,61].

These scaffolds frequently serve as ATP-mimetic motifs capable of occupying kinase active sites and disrupting oncogenic signaling pathways. Consequently, they have become key structural elements in inhibitors targeting EGFR, VEGFR, BRAF, CDKs, JAK kinases, and numerous other therapeutically relevant proteins [43,62]. Many clinically approved targeted therapies are based on heterocyclic frameworks, highlighting their central role in modern precision oncology (Table 1) [9,43,44]. Beyond their therapeutic application, several heterocycle-based inhibitors have served as molecular templates for radiopharmaceuticals. For example, radiolabeled PARP inhibitors, EGFR-targeting tracers, and prostate-specific membrane antigen (PSMA)-directed small molecules present how scaffold optimization can simultaneously support molecular imaging and targeted radionuclide therapy [26,27,63,64].

Pyrazoles are particularly valued for their ability to participate in hydrogen-bonding interactions within kinase hinge regions, resulting in potent and selective inhibition of signaling pathways involved in tumor proliferation and angiogenesis [34]. Clinically, pyrazole-containing molecules have demonstrated considerable success as kinase inhibitors, exemplified by crizotinib and related targeted therapies, highlighting the translational potential of this scaffold for precision oncology [61,66].

Pyridazines offer similar advantages while providing opportunities for structural diversification and optimization of pharmacokinetic properties. Although relatively few pyridazine-derived anticancer drugs have reached clinical application compared with quinazolines, this scaffold has demonstrated considerable promise in VEGFR, FGFR, and CDK inhibition, making it an attractive platform for future targeted therapeutics [67,68]. Although clinical translation remains more limited than for quinazolines or pyrimidines, pyridazine derivatives continue to attract considerable interest as emerging kinase-directed scaffolds.

Quinazolines have achieved exceptional clinical success as the core structures of several generations of epidermal growth factor receptor inhibitors and continue to serve as privileged scaffolds for targeted drug discovery [43,62]. Structure–activity relationship studies of quinazolinone revealed that positions 2, 6, and 8 of the ring system are significant due to different pharmacological activities [42]. The clinical evolution of quinazoline-based EGFR inhibitors provides one of the best examples of effective scaffold optimization. Progressive modification of substituents around the quinazoline core resulted in successive generations of inhibitors, including Gefitinib, Erlotinib, Afatinib, and Osimertinib, with improvements in potency, selectivity, and activity against resistance-associated EGFR mutations [44,62,71]. This progression shows one of the clearest examples of successful scaffold optimization in medicinal chemistry. The same scaffold has also facilitated the development of radiolabeled EGFR-targeting probes for non-invasive assessment of receptor expression and treatment response, further illustrating the versatility of quinazoline chemistry beyond conventional kinase inhibition.

Pyrimidine derivatives are represented among numerous clinically approved kinase inhibitors, including CDK4/6 inhibitors such as Abemaciclib and Ribociclib. Their structural similarity to naturally occurring nucleobases contributes to efficient recognition by ATP-binding enzymes, facilitating selective kinase inhibition [35,70]. The ability of pyrimidines to mimic endogenous nucleobases contributes to their exceptional versatility across multiple kinase families.

The radiotheranostic relevance of heterocyclic scaffolds derives largely from their target specificity and synthetic adaptability. Many heterocycle-based inhibitors can be modified with minimal disruption of biological activity, enabling incorporation of radionuclides or chelating functionalities for molecular imaging applications [75,76]. Radiolabeled kinase inhibitors and PARP-targeted tracers exemplify this strategy, demonstrating how scaffold architecture can support both therapeutic and diagnostic functions [61]. Similarly, clinically established radiotheranostic platforms targeting PSMA, SSTR, and fibroblast activation protein demonstrate how rational scaffold selection, combined with appropriate chelator and radionuclide pairing, enables successful translation into precision oncology. In contrast to TSCs, heterocyclic systems typically rely on target-driven accumulation rather than intrinsic metal-binding properties, but their exceptional selectivity often makes them highly suitable for companion diagnostic usage.

### 3.3. Metal-Based Scaffolds

Metal-containing compounds continue to play a significant role in oncology despite the growing dominance of targeted therapies. Although platinum-based drugs remain among the most widely used anticancer agents, increasing attention has shifted toward alternative transition-metal scaffolds capable of overcoming resistance mechanisms and providing novel modes of action. Unlike classical organic scaffolds, metal-containing compounds introduce unique coordination geometries and redox properties that substantially expand the available chemical space for anticancer drug discovery.

Copper, ruthenium, and gold complexes have emerged as particularly promising candidates. Ruthenium compounds possess favorable coordination chemistry and may mimic certain biological properties of iron, facilitating interactions with DNA, proteins, and redox-regulating systems [77,78]. Gold complexes frequently target thiol-containing enzymes such as thioredoxin reductase, disrupting cellular redox homeostasis and promoting apoptosis [79]. Copper complexes often exploit altered metal metabolism in tumors while simultaneously generating cytotoxic oxidative stress [12,65]. Among these, copper-based systems are of particular interest because isotopes such as ^64Cu and ^67Cu constitute one of the few true diagnostic–therapeutic radionuclide pairs suitable for radiotheranostic applications.

The relevance of metal-based scaffolds to radiotheranostics is evident. Their coordination environments can frequently be adapted for incorporation of radiometals, while their biological mechanisms often complement radiation-induced cytotoxicity. As a result, metal-based architectures provide a valuable interface between classical coordination chemistry and modern nuclear medicine.

### 3.4. Hybrid Scaffolds

The growing complexity of cancer biology has stimulated the development of hybrid molecules capable of simultaneously modulating multiple therapeutic targets. Hybrid scaffolds integrate distinct pharmacophoric elements within a single molecular framework, creating multifunctional agents that may achieve superior efficacy compared with individual components administered separately [29]. Examples include PARP-topoisomerase hybrids, kinase-epigenetic modulator conjugates, DNA-interacting redox-active systems, and TSC-heterocycle combinations. Such architectures can influence multiple signaling pathways, enhance anticancer activity, and reduce the likelihood of therapeutic resistance. Importantly, hybridization also provides opportunities to integrate targeting motifs, radionuclide-binding domains, and therapeutic pharmacophores within a unified scaffold [74].

From a radiotheranostic perspective, hybrid systems may represent one of the most promising directions in future drug discovery. Their modular architecture enables simultaneous optimization of biological activity, target specificity, pharmacokinetics, and radionuclide compatibility. Nevertheless, despite their considerable promise, hybrid scaffold systems remain largely at the preclinical stage, and further optimization of pharmacokinetics, safety, manufacturing, and clinical translation is required before routine application.

### 3.5. Emerging Scaffolds

Several emerging scaffold families are attracting increasing attention because of their potential to expand the capabilities of anticancer and radiotheranostic drug design. Among these are PROTAC-derived molecular architectures, boron-containing compounds, macrocyclic systems, and small-molecule precursors for nanotheranostic applications [80].

PROTAC-based scaffolds introduce a fundamentally different therapeutic paradigm by promoting targeted protein degradation rather than conventional inhibition. Their modular design may also promote radiolabeling strategies for imaging protein degradation kinetics and treatment response [80,81]. Boron-rich compounds, particularly carboranes, are gaining importance through their association with boron neutron capture therapy and their unique physicochemical characteristics [82]. Macrocyclic structures offer exceptional binding affinity and stability, making them attractive candidates for radiometal coordination and targeted radionuclide delivery [81]. At present, most of these emerging scaffold classes remain in the preclinical phase; however, they indicate future directions in scaffold-guided radiotheranostic design and may provide the basis for next-generation precision oncology platforms.

## 4. Mechanistic Basis

While chemical scaffolds differ substantially in structure and primary biological targets, many ultimately converge on a limited number of cellular processes that govern tumor survival and treatment response. Understanding these shared mechanistic pathways is essential not only for rational anticancer drug design but also for identifying scaffold classes suitable for radiotheranostic translation. Importantly, several mechanisms exploited by contemporary anticancer agents closely parallel those responsible for radionuclide-induced cytotoxicity, creating opportunities for synergistic therapeutic effects.

Rather than acting through entirely independent pathways, many scaffolds discussed in this review influence DNA integrity, redox homeostasis, cell-cycle regulation, and oncogenic signaling networks. These same biological systems are frequently involved in determining tumor sensitivity to ionizing radiation. Consequently, scaffold-mediated anticancer activity and radionuclide therapy often exhibit significant mechanistic overlap, providing a biological rationale for the integrated radiotheranostic strategies.

### 4.1. DNA Damage and DNA Repair Modulation

Maintenance of genomic integrity is critical for cancer cell survival, particularly in rapidly proliferating tumors characterized by elevated replication stress and genomic instability. Numerous anticancer scaffolds exert their activity by directly or indirectly inducing DNA damage or by interfering with DNA repair pathways responsible for maintaining genomic stability.

Several heterocyclic and hybrid scaffolds target enzymes involved in DNA processing, including topoisomerases and poly(ADP-ribose) polymerases (PARPs). Topoisomerase inhibition results in accumulation of DNA strand breaks, disruption of replication fork progression, and activation of DNA damage response pathways. Similarly, PARP inhibition impairs repair of single-strand DNA lesions, promoting the formation of double-strand breaks during replication and ultimately triggering cell death [83]. These mechanisms are highly relevant to radiotheranostics because ionizing radiation exerts much of its cytotoxic effect through DNA damage, particularly double-strand breaks. Tumor cells with compromised DNA repair capacity are therefore significantly more sensitive to radiation-induced injury. As a result, scaffolds capable of interfering with DNA repair pathways may function as molecular radiosensitizers, enhancing the efficacy of targeted radionuclide therapy [83]. The success of PARP inhibitors in oncology illustrates this principle particularly well. Beyond their direct therapeutic activity, PARP-targeted scaffolds have become attractive candidates for radiolabeled imaging agents capable of visualizing DNA repair activity and identifying patients likely to benefit from DNA damage–directed therapies. Several PARP-targeted radiopharmaceuticals have already entered preclinical and early clinical evaluation, illustrating the clinically applicable translation of scaffold-based medicinal chemistry into molecular imaging applications.

### 4.2. Redox Modulation and Oxidative Stress

Disruption of redox homeostasis represents another major mechanism shared by multiple anticancer scaffold classes. Cancer cells typically exhibit elevated levels of reactive oxygen species as a consequence of metabolic reprogramming, mitochondrial dysfunction, oncogenic signaling, and rapid proliferation. Although moderate ROS levels may support tumor progression, excessive oxidative stress overwhelms cellular defense mechanisms and promotes cell death [57]. Thiosemicarbazones and numerous metal-based compounds are particularly effective modulators of redox biology [55,65]. Through metal chelation and redox cycling, these scaffolds generate ROS that damage proteins, lipids, mitochondria, and nucleic acids. Similar mechanisms contribute to the activity of several copper-, ruthenium-, and gold-containing anticancer agents [37].

The importance of oxidative stress extends beyond conventional chemotherapy. Ionizing radiation also induces cellular injury primarily through generation of free radicals and ROS. Consequently, scaffolds that enhance oxidative stress may amplify radiation-mediated cytotoxicity. This mechanistic convergence is especially relevant for radiotheranostics because it enables simultaneous exploitation of biochemical and radiological mechanisms within a single therapeutic strategy. In this context, redox-active scaffolds may function as intrinsic radiosensitizers, lowering the threshold required for radiation-induced cell death and potentially improving therapeutic outcomes.

### 4.3. Inhibition of Nucleotide Biosynthesis and Cell-Cycle Regulation

Cell proliferation requires continuous synthesis of nucleotides to support DNA replication and repair. Disruption of nucleotide metabolism therefore represents an effective strategy for inhibiting tumor growth. Among the scaffold classes discussed in this review, thiosemicarbazones are particularly notable for their ability to inhibit ribonucleotide reductase, the rate-limiting enzyme responsible for de novo deoxyribonucleotide synthesis. By interfering with iron-dependent catalytic processes, TSCs reduce intracellular nucleotide availability, suppress DNA replication, and induce cell-cycle arrest [84,85].

Cell-cycle perturbation has important implications for radiotherapy and radionuclide therapy because cellular radiosensitivity varies throughout the cell cycle. Cells are generally most radiosensitive during the G2/M phases, whereas late S-phase cells are relatively radioresistant. Thus, scaffold-induced synchronization of tumor populations within radiosensitive phases may enhance the effectiveness of radiation-based treatments [35]. Consequently, scaffold-induced synchronization of tumor populations within radiosensitive phases may enhance the effectiveness of radiation-based treatments.

### 4.4. Modulation of Oncogenic Signaling Networks

Targeted inhibition of oncogenic signaling pathways remains one of the most favorable strategies in modern oncology [1,2,35]. Heterocyclic scaffolds have played a particularly important role in this area because their structural features allow precise interactions with enzyme active sites and receptor binding domains [36]. Pyrazoles, pyridazines, quinazolines, and related heterocyclic systems serve as core structures for numerous kinase inhibitors targeting EGFR, VEGFR, v-Raf murine sarcoma viral oncogene homolog B1 (BRAF), CDKs, Janus kinases (JAK), and other signaling proteins involved in proliferation, angiogenesis, survival, and metastasis. By disrupting these pathways, such compounds suppress tumor growth and may restore sensitivity to other therapeutic modalities [35,36].

From a radiotheranostic perspective, signaling pathway modulation offers an additional advantage: the same molecular targets exploited therapeutically can often be visualized through molecular imaging. Radiolabeled kinase inhibitors and receptor-targeted tracers provide non-invasive information regarding target expression, treatment response, and resistance mechanisms. Clinically established examples include PSMA- and somatostatin receptor-targeted radiopharmaceuticals, whereas FAP-targeting tracers represent rapidly emerging platforms that further illustrate the efficient integration of molecular targeting with diagnostic imaging and targeted radionuclide therapy. This dual functionality exemplifies the close relationship between scaffold design and radiotheranostic development.

### 4.5. Mechanistic Convergence with Radionuclide Therapy

Although anticancer drugs and radionuclide therapies are frequently considered distinct treatment modalities, their biological effects often intersect at several critical points. DNA damage, oxidative stress, cell-cycle disruption, and impairment of repair pathways are common consequences of both scaffold-mediated pharmacological intervention and ionizing radiation exposure. Importantly, the relative contribution of these mechanisms may differ depending on the physical characteristics of the radionuclide employed. β-emitters primarily induce DNA damage through crossfire effects, α-particles generate highly localized high-linear-energy-transfer (LET) damage, whereas Auger electron emitters require close proximity to cellular DNA to achieve maximal therapeutic efficacy. This mechanistic convergence provides a strong rationale for integrating medicinal chemistry and radiotheranostic design. Scaffolds that inhibit DNA repair may increase susceptibility to radiation-induced damage. Redox-active compounds can amplify ROS generation following radionuclide decay. Targeted inhibitors may facilitate selective delivery of radiation to biologically relevant tumor subpopulations. Hybrid architectures may combine these advantages within a single molecular entity.

**Figure 6:**
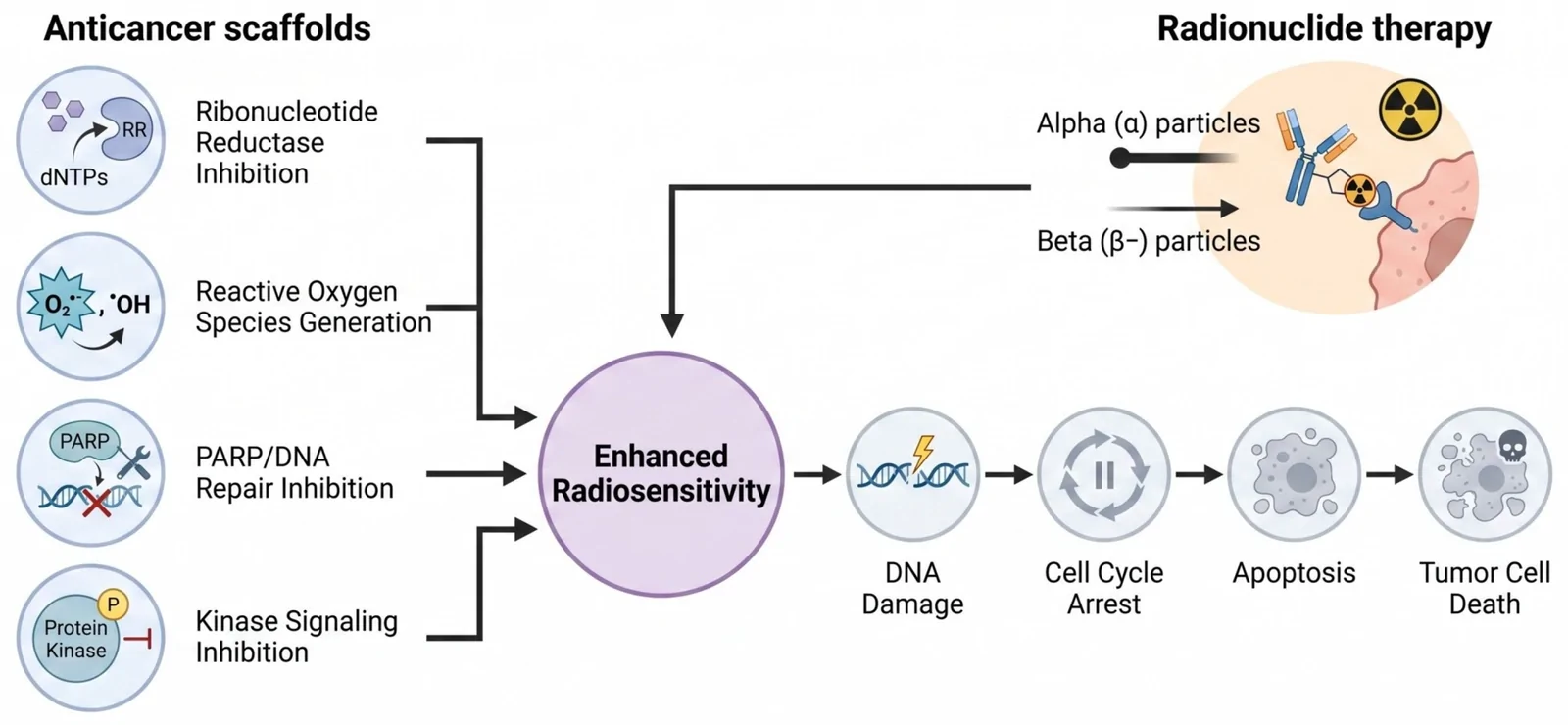
Mechanistic convergence between anticancer scaffold activity and targeted radionuclide therapy. Representative anticancer scaffold classes influence key biological pathways including ribonucleotide reductase inhibition, reactive oxygen species generation, PARP/DNA repair inhibition, and kinase signaling modulation. These mechanisms converge with radiation-induced cellular injury to enhance radiosensitivity, DNA damage, cell-cycle arrest, apoptosis, and tumor cell death. Created in BioRender. Rusek, M. (2026) https://BioRender.com/x5y4rgv (accessed on 28 July 2026).

Moreover, effective radiotheranostic implementation depends not only on radionuclide selection but also on the biological properties of the underlying scaffold. Consequently, optimal scaffold selection should be considered together with radionuclide properties, biological target characteristics, and clinical indications to maximize therapeutic efficacy while minimizing normal-tissue toxicity. The most effective platforms are likely to be those capable of simultaneously providing target specificity, intrinsic anticancer activity, and enhancement of radiation-mediated cytotoxicity. Therefore, these observations highlight that the relationship between chemical scaffolds and radiotheranostics extends far beyond simple radiolabeling. Instead, scaffold architecture frequently determines the biological context in which radionuclide therapy operates, influencing target engagement, radiosensitivity, and therapeutic response. Understanding these mechanistic interactions is therefore essential for the rational development of clinically translatable radiotheranostic agents capable of maximizing both diagnostic and therapeutic performance. The mechanistic convergence between scaffold activity and radionuclide therapy is illustrated in Figure 6.

## 5. Structural Determinants of Radiotheranostic Translation

Building upon the mechanistic principles discussed above, efficient radiotheranostic translation ultimately depends on a series of structural characteristics that determine whether a scaffold can progress from a biologically active molecule to a clinically useful theranostic platform. The rapid expansion of radiotheranostics has transformed the way molecular imaging and cancer therapy are conceptualized and implemented. Traditionally, the development of anticancer drugs and radiopharmaceuticals proceeded along largely independent trajectories, with medicinal chemistry focusing on biological activity and nuclear medicine emphasizing target visualization and radionuclide delivery. However, successful radiotheranostic agents emerge from molecular architectures that simultaneously satisfy the requirements of both disciplines.

Importantly, potent anticancer activity alone does not guarantee favorable radiotheranostic translation. Instead, clinically useful platforms must simultaneously satisfy structural, pharmacological, physicochemical, and radiochemical requirements governing target engagement, radionuclide stability, biodistribution, and therapeutic efficacy. Together, these characteristics constitute the structural determinants of radiotheranostic translation discussed below.

### 5.1. Target Affinity and Molecular Recognition

The most fundamental requirement for any radiotheranostic platform is selective interaction with a biologically relevant target. High target affinity promotes tumor accumulation, improves imaging contrast, and increases therapeutic efficacy while minimizing exposure of healthy tissues. The importance of this principle is evident in clinically successful radiotheranostic systems targeting prostate-specific membrane antigen, somatostatin receptors, and fibroblast activation protein [18,19,86,87]. These examples also demonstrate that target biology ultimately determines whether a scaffold is suitable for diagnostic imaging, radionuclide therapy, or true theranostic pairing. From a scaffold perspective, molecular recognition is largely determined by the spatial arrangement of pharmacophoric elements and the ability to establish productive interactions with target proteins. Heterocyclic scaffolds are particularly advantageous because their electronic properties and hydrogen-bonding capabilities support highly specific interactions with kinase active sites, DNA repair enzymes, and receptor proteins. Similarly, hybrid architectures can combine multiple recognition motifs within a single molecular framework, increasing target selectivity and biological complexity [88,89].

For radiotheranostic applications, preservation of target affinity following radiolabeling is especially important. Structural modifications required for radionuclide incorporation should ideally occur at positions that do not interfere with target recognition. Scaffold classes possessing chemically accessible functionalization sites therefore offer significant advantages during radiopharmaceutical optimization [20,88,89].

### 5.2. Metal-Chelating Pharmacophores

A distinguishing feature of many successful radiotheranostic scaffolds is their capacity to support stable radionuclide incorporation. The ability to coordinate diagnostic and therapeutic isotopes directly influences radiochemical stability, biodistribution, and safety. Scaffolds possessing intrinsic metal-chelating functionality occupy a particularly advantageous position in this regard. Thiosemicarbazones represent a prominent example because their NNS donor system naturally coordinates transition metals, including copper isotopes relevant to theranostic applications. In contrast, many heterocyclic inhibitors require attachment of additional chelating moieties such as DOTA, NOTA, or related structures to enable radiometal incorporation [90,91].

The choice of radionuclide further influences scaffold requirements. Diagnostic isotopes used in PET imaging, such as ^18F, ^68Ga, and ^64Cu, require efficient labeling procedures and sufficient in vivo stability to permit accurate imaging [13,14]. Therapeutic radionuclides including ^67Cu, ^177Lu, and ^225Ac impose even stricter requirements because radionuclide dissociation may result in off-target toxicity and reduced treatment efficacy [13,90]. Consequently, scaffold architecture plays a central role in determining radionuclide compatibility. Structures capable of maintaining stable radionuclide coordination under physiological conditions possess a substantial translational advantage. Beyond stable complex formation, practical radionuclide selection must also consider isotope availability, production logistics, regulatory accessibility, and compatibility between radionuclide half-life and the biological kinetics of the scaffold.

### 5.3. Structural Modularity and Synthetic Accessibility

Radiotheranostic optimization frequently requires extensive structural modification to balance biological activity, pharmacokinetics, imaging performance, and therapeutic efficacy. Consequently, modular scaffolds possessing chemically accessible functionalization sites provide a substantial translational advantage by enabling systematic optimization without disrupting biological activity [37,38]. Therefore, scaffold modularity represents a critical determinant of translational success. Modular scaffolds provide chemically accessible positions for incorporation of targeting ligands, pharmacokinetic modifiers, fluorescent probes, chelators, and radionuclides. Thiosemicarbazones exemplify this principle through the ability to modify both the carbonyl precursor and terminal nitrogen substituents while preserving metal-binding functionality [55,65]. Similarly, heterocyclic scaffolds often contain multiple positions amenable to substitution, facilitating fine-tuning of target affinity and radiopharmaceutical behavior [35,36].

Synthetic accessibility also plays an important practical role. Complex molecular architectures may demonstrate impressive biological activity but encounter significant barriers to large-scale synthesis, regulatory development, and clinical translation [40,92]. Scaffolds supported by robust and versatile synthetic methodologies are therefore more likely to progress successfully from discovery to clinical application. Consequently, synthetic simplicity should be regarded as a translational advantage rather than merely a synthetic convenience.

### 5.4. Pharmacokinetic Tunability

The biological performance of radiotheranostic agents depends not only on target affinity but also on favorable pharmacokinetic behavior. Optimal compounds must achieve sufficient tumor accumulation while simultaneously minimizing retention in non-target tissues. Scaffold architecture strongly influences parameters such as lipophilicity, membrane permeability, plasma protein binding, metabolic stability, and clearance pathways. Excessively lipophilic molecules may exhibit undesirable hepatic accumulation, whereas highly hydrophilic compounds may suffer from poor cellular uptake. The ability to adjust these properties through rational scaffold modification is therefore highly advantageous [39,40].

In addition, pharmacokinetic optimization often differs between diagnostic and therapeutic applications. Imaging agents require rapid target localization and efficient background clearance to maximize image contrast, whereas therapeutic agents may benefit from prolonged tumor retention. Scaffold classes capable of accommodating both objectives through controlled structural modification are particularly attractive for radiotheranostics.

### 5.5. Biological Radiosensitization Potential

One of the most intriguing determinants of radiotheranostic suitability extends beyond radionuclide delivery itself. Certain scaffolds possess intrinsic biological properties capable of enhancing radiation-induced cytotoxicity, thereby functioning as molecular radiosensitizers [11,55]. Redox-active systems such as thiosemicarbazones and selected metal complexes illustrate this concept particularly well. Through generation of reactive oxygen species and disruption of cellular antioxidant defenses, these compounds amplify oxidative stress within tumor cells. Because ionizing radiation also produces cytotoxic free radicals, simultaneous activation of both mechanisms may generate synergistic therapeutic effects [37,65].

Similarly, scaffolds that interfere with DNA repair pathways, cell-cycle checkpoints, or stress-response signaling networks may increase susceptibility to radiation-induced damage. Such mechanisms suggest that the ideal radiotheranostic scaffold is not merely a radionuclide carrier but an active participant in the therapeutic process [83]. This concept represents an important evolution in radiotheranostic design. Future agents are increasingly likely to combine radionuclide delivery with biologically complementary mechanisms that enhance treatment efficacy while maintaining molecular specificity. This concept shifts scaffold design from passive radionuclide carriers toward biologically active therapeutic partners capable of enhancing radionuclide efficacy through complementary molecular mechanisms.

### 5.6. Hybridization Potential and Multifunctionality

Modern radiotheranostics increasingly favors multifunctional molecular architectures capable of performing several tasks simultaneously [37,83]. In this context, scaffold compatibility with molecular hybridization becomes a critical determinant of future applicability. Hybrid scaffolds enable integration of targeting motifs, therapeutic pharmacophores, chelating domains, and imaging functionalities within a single molecular framework [74]. Such systems may support tumor targeting, therapeutic activity, diagnostic imaging, and treatment monitoring simultaneously [37]. Examples include kinase inhibitor–radiometal conjugates, PARP-targeted imaging agents and TSC–heterocycle hybrids combining metal coordination with pathway-specific biological activity. The growing success of these approaches suggests that multifunctionality may become a defining characteristic of next generation radiotheranostic platforms [11,37,55]. Scaffold classes capable of supporting such integration possess substantial advantages over more structurally restrictive systems and are likely to play an increasingly important role in future precision oncology strategies.

### 5.7. A Framework for Evaluating Radiotheranostic Suitability

Collectively, the characteristics discussed above suggest that radiotheranostic translation can be evaluated using a scaffold-centered framework encompassing six major determinants such as target affinity and selectivity, metal coordination and radionuclide compatibility, structural modularity, pharmacokinetic tunability, radiosensitization potential, and hybridization capability. These determinants provide a practical basis for comparing scaffold classes and identifying promising candidates for future development. Structural determinants are summarized in Table 2.

When considered together, these parameters reveal why certain scaffold classes repeatedly emerge in successful radiotheranostic systems. Rather than representing isolated examples, thiosemicarbazones, heterocycles, metal-based compounds, and hybrid architectures share structural characteristics that facilitate the integration of medicinal chemistry and nuclear medicine. Understanding these common principles may ultimately guide the rational design of next-generation agents capable of combining molecular targeting, diagnostic imaging, and radionuclide therapy within a unified precision oncology framework.

### 5.8. Comparative Assessment, Challenges and Future Directions

Although numerous chemical scaffolds exhibit promising anticancer activity, their suitability for radiotheranostic applications varies considerably. This variability arises from differences in target specificity, radionuclide compatibility, structural flexibility, pharmacokinetic behavior, and the ability to support multifunctional design strategies. Thus, a comparative assessment of scaffold classes may provide valuable guidance for future radiotheranostic translation.

Among the scaffold families discussed in this review, thiosemicarbazones represent one of the most versatile platforms. Their unique combination of intrinsic metal-chelating capacity, redox activity, and structural adaptability enables simultaneous exploitation of pharmacological and radiopharmaceutical properties. Unlike many conventional targeted agents, TSCs naturally accommodate transition metals and therefore possess a distinct advantage in the development of copper-based theranostic systems. In addition, their ability to modulate oxidative stress and nucleotide biosynthesis may enhance radiation-induced cytotoxicity, providing opportunities for synergistic therapeutic effects [10,11]. However, their biological complexity and metal-dependent pharmacology require careful optimization before broad clinical implementation.

Heterocyclic scaffolds, including pyrazoles, pyridazines, and quinazolines, remain dominant in targeted anticancer drug discovery because of their exceptional target affinity and selectivity. These systems have demonstrated remarkable success in kinase inhibition and molecularly targeted therapies [1,2,35,36]. Their primary radiotheranostic advantage lies in the ability to generate highly specific imaging agents and companion diagnostics through relatively minor structural modifications [37]. However, unlike TSCs, most heterocyclic scaffolds require additional chelating moieties for radiometal incorporation, which may complicate optimization and influence pharmacokinetic behavior [37,38]. Nevertheless, the absence of intrinsic metal-binding capability usually necessitates additional chelator conjugation, increasing structural complexity.

Metal-based scaffolds occupy an intermediate position between classical anticancer agents and radiopharmaceutical platforms [13,14]. Their coordination chemistry facilitates radionuclide incorporation and supports mechanisms complementary to radiation-induced cellular damage. However, concerns regarding toxicity, radiochemical stability, and off-target accumulation continue to limit clinical translation [14,93].

Hybrid scaffolds appear particularly attractive from a radiotheranostic perspective because they integrate multiple desirable properties within a single molecular architecture. By combining targeting motifs, biologically active pharmacophores, and radionuclide-compatible functionalities, hybrid systems offer opportunities for simultaneous imaging, therapy, and treatment monitoring [94]. Their modularity also enables optimization of pharmacokinetic and biological properties, making them highly adaptable to diverse oncological applications [94]. However, increasing molecular complexity may adversely affect manufacturability, pharmacokinetics, and regulatory development.

Taken together, these observations suggest that no single scaffold class is universally superior for all radiotheranostic applications. Instead, successful scaffold selection should be guided by the intended clinical objective, target biology, radionuclide requirements, and desired mechanism of action. Nevertheless, scaffold classes combining target specificity, radionuclide compatibility, structural flexibility, and intrinsic therapeutic activity are likely to play the most prominent role in the next generation of precision oncology agents. Future scaffold selection is therefore likely to become increasingly application-driven, with different structural platforms being preferred for PET imaging, targeted radionuclide therapy, companion diagnostics, or integrated theranostic applications rather than a universal scaffold suitable for all purposes.

Despite substantial progress in radiotheranostic research, the successful translation of anticancer scaffolds into clinically useful imaging and therapeutic agents remain associated with several challenges. One of the most common limitations is the potential loss of biological activity following radiolabeling. Structural modifications required for radionuclide incorporation may alter target affinity, binding kinetics, or pharmacokinetic behavior, thereby reducing therapeutic performance. Radiometal-based systems present additional challenges related to complex stability and radionuclide retention. Inadequate chelation may result in radionuclide dissociation, non-specific tissue accumulation, and increased toxicity. Furthermore, differences in physical half-life, radiation characteristics, and production availability among radionuclides complicate scaffold selection and optimization [91].

Pharmacokinetic considerations also remain critical. While prolonged tumor retention may enhance therapeutic efficacy, excessive accumulation in healthy tissues can increase radiation exposure and adverse effects. Achieving an optimal balance between target uptake and background clearance therefore represents a major objective during radiotheranostic development [37,75,83].

Manufacturing and regulatory considerations further influence translational feasibility. Complex multifunctional scaffolds often require challenging synthetic routes, specialized radiochemistry infrastructure, and extensive quality-control procedures. These factors may significantly increase costs and limit large-scale clinical implementation [17,95]. Additional barriers include patient selection, intertumoral biological heterogeneity, dosimetric optimization, and the need for prospective clinical validation. Addressing these challenges will require close collaboration among medicinal chemists, radiochemists, nuclear medicine specialists, pharmacologists, and clinical oncologists.

Future progress in scaffold-based radiotheranostics will require not only the identification of biologically effective scaffold architectures but also careful consideration of radiochemical stability, pharmacokinetic optimization, manufacturability, and clinical practicality. Ultimately, the future of scaffold-based radiotheranostics will depend on the successful integration of medicinal chemistry, radiochemistry, molecular imaging, radiation biology, and clinical oncology into unified translational strategies.

## 6. Future Perspectives

The continued convergence of medicinal chemistry, molecular imaging, and targeted radionuclide therapy is reshaping the landscape of anticancer drug discovery. While considerable progress has been achieved through the optimization of individual pharmacophores and radiopharmaceutical platforms, future advances are likely to emerge from increasingly integrated approaches that combine biological targeting, therapeutic activity, and diagnostic functionality within unified molecular architectures. In this evolving environment, chemical scaffolds will play a central role not only as structural frameworks but also as determinants of translational potential. Future success will increasingly depend on designing scaffold architectures with clinical translation in mind from the earliest stages of medicinal chemistry rather than adapting biologically active molecules for radiotheranostic use at later stages of development.

One of the most promising directions involves the development of multifunctional hybrid scaffolds capable of simultaneously engaging multiple oncogenic pathways while supporting radiotheranostic applications. Cancer progression is driven by complex and interconnected signaling networks, making single target approaches increasingly insufficient in many clinical settings. Hybrid architectures provide opportunities to integrate complementary mechanisms within a single molecule, potentially enhancing efficacy while reducing the likelihood of resistance development. However, increasing molecular complexity will also require careful optimization of pharmacokinetic behavior, manufacturability, and regulatory feasibility before routine clinical implementation can be achieved.

Among the scaffold classes discussed in this review, thiosemicarbazones appear particularly well positioned for such applications. developments. Their intrinsic metal-chelating properties, redox activity, and structural adaptability provide a versatile foundation for the construction of multifunctional agents. Future designs may combine TSC pharmacophores with kinase-targeting heterocycles, DNA repair inhibitors, or receptor-directed ligands to generate compounds capable of simultaneous molecular inhibition and radionuclide delivery. Such systems could exploit both pharmacological and radiological mechanisms of action, offering a level of therapeutic integration that extends beyond current radiopharmaceutical strategies [11,96].

The increasing importance of copper-based theranostic systems is likely to further stimulate interest in scaffold classes compatible with radiometal coordination. The complementary properties of ^64Cu and ^67Cu provide a particularly attractive framework for paired diagnostic and therapeutic applications [14]. The emergence of additional matched radionuclide pairs is expected to further broaden the range of scaffold architectures suitable for true theranostic applications. Scaffold architectures capable of maintaining stable copper coordination while preserving biological activity may therefore become increasingly valuable in future radiotheranostic development [13,14]. In this context, the distinction between anticancer drug and radiopharmaceutical may gradually become less pronounced, with many next-generation agents designed from the outset to fulfill both functions [12,19,97].

Another emerging trend is the expansion of companion diagnostic strategies. Molecular imaging is increasingly used not only to detect disease but also to guide patient selection, monitor therapeutic response, and identify mechanisms of resistance. Consequently, scaffolds that can be readily adapted into imaging probes are likely to play a growing role in precision oncology. The ability to visualize target expression non-invasively before initiating therapy may improve treatment selection and reduce unnecessary exposure to ineffective interventions [5,24,25,27,98].

Advances in artificial intelligence and computational drug design are also expected to accelerate scaffold discovery and optimization. Machine learning algorithms can now analyze large chemical and biological datasets to identify relationships between molecular structure and biological activity, predict pharmacokinetic properties, and suggest novel scaffold architectures [5,99,100]. As radiotheranostic datasets become increasingly available, AI-assisted approaches may promote the identification of scaffold characteristics associated with successful radionuclide incorporation, favorable biodistribution, and therapeutic efficacy. Such technologies could significantly shorten development timelines and improve the efficiency of lead optimization [99,100,101]. Beyond lead discovery, AI may also support patient stratification, prediction of treatment response, and optimization of radionuclide-scaffold combinations using multimodal clinical datasets.

The future of scaffold-based radiotheranostics will also benefit from improvements in radiochemistry and radionuclide availability. Expanding access to therapeutic isotopes, advances in chelator design, and the development of novel radiolabeling methodologies will increase the range of scaffold classes suitable for clinical translation. Simultaneously, innovations in click chemistry, late-stage functionalization, and modular synthetic approaches will support rapid incorporation of radionuclides into biologically active molecules without extensive redesigning the parent scaffold [12,17,19,95,97]. These advances may substantially reduce the time required to translate promising medicinal chemistry leads into clinically applicable radiopharmaceuticals.

An additional area of growing interest involves the integration of radiotheranostics with other treatment modalities, including immunotherapy and targeted protein degradation. Such combinations may ultimately enable personalized multimodal treatment strategies in which molecular imaging guides the selection and monitoring of complementary therapeutic interventions. Radiotherapy-induced immunogenic cell death may enhance antitumor immune responses, while radiolabeled molecular degraders could provide entirely new approaches to target validation and treatment monitoring [23,87]. Scaffold architectures capable of supporting such multifunctional strategies may represent an important frontier in future oncology research [17,95].

The most successful next generation radiotheranostic agents are unlikely to arise from simple radiolabeling of existing anticancer drugs. Instead, they will emerge from scaffold-centered design strategies in which medicinal chemistry, radiochemistry, molecular imaging, and radiation biology are integrated from the earliest stages of drug discovery.

## 7. Conclusions

Chemical scaffolds constitute the molecular foundation of modern anticancer drug discovery and increasingly influence the development of radiotheranostic agents. While traditionally viewed as structural frameworks supporting biological activity, scaffolds are now recognized as critical determinants of target recognition, pharmacokinetic behavior, radionuclide compatibility, and translational potential. Scaffold selection has become an essential consideration not only in medicinal chemistry but also in the design of next-generation molecular imaging and targeted radionuclide therapy platforms.

This review highlights how diverse scaffold classes, including thiosemicarbazones, heterocyclic systems, metal-based compounds, hybrid architectures, and emerging multifunctional platforms contribute to contemporary oncology through distinct complementary mechanisms of action. Although these scaffold classes differ substantially in structure and primary molecular targets, many of these scaffolds converge on common biological pathways involving DNA damage, oxidative stress, nucleotide biosynthesis, and oncogenic signaling pathways. In addition, these same pathways frequently determine tumor sensitivity to ionizing radiation, creating opportunities for synergistic interactions between pharmacological and radiological therapies.

A central theme emerging from this analysis is that successful radiotheranostic translation depends on a specific combination of structural and functional characteristics. High target affinity, radionuclide compatibility, modularity, pharmacokinetic tunability, radiosensitization potential, and hybridization capability collectively define the suitability of a scaffold for radiotheranostic development. Scaffold classes possessing several of these attributes simultaneously are particularly attractive candidates for future precision oncology applications. Among the scaffold classes discussed, thiosemicarbazones illustrate particularly well how intrinsic metal-chelating capability, biological activity, and radionuclide compatibility may converge within a single molecular framework, although further optimization and clinical validation remain necessary.

As radiotheranostics continues to evolve, the boundaries between medicinal chemistry, molecular imaging, and targeted radionuclide therapy are becoming increasingly interconnected. Continued advances in precision oncology will depend not only on the discovery of novel bioactive molecules but also on the rational identification of scaffold architectures inherently compatible with radiotheranostic translation. In this context, scaffold selection should no longer be regarded solely as the starting point of medicinal chemistry but rather as the central design principle connecting molecular recognition, targeted therapy, diagnostic imaging, and precision radiotheranostics. I anticipate that future breakthroughs in radiotheranostics will arise not from individual technological advances but from the successful integration of scaffold-guided medicinal chemistry with radiochemistry, molecular imaging, and translational oncology.

## Figures and Tables

**Table 1:** Comparative overview of representative anticancer scaffold classes and their relevance for radiotheranostic development.

Scaffold Class	Representative Drugs/Compounds	Primary Molecular Target(s)	Representative Potency (IC_50_)	Key Structural Features	Major Biological Mechanism(s)	Radiotheranostic Potential *	Ref.
**Thiosemicarbazones**	Triapine; Dp44mT	RR; metal-dependent pathways	Triapine: assay-dependent inhibition of RNR; Dp44mT: target- and assay-dependent	N,S-donor pharmacophore; intrinsic metal-chelating ability; highly modular scaffold	RR inhibition, ROS generation, metal chelation	Excellent– intrinsic coordination of radiometals, radiosensitization	[55,65]
**Pyrazoles**	Crizotinib	ALK, ROS1, MET	ALK: 20 nM; MET: 8 nM	Compact heterocyclic core; high synthetic versatility; extensive substitution possibilities	Kinase inhibition	Moderate–requires linker/chelator incorporation	[35,36,66]
**Pyridazines**	Representative VEGFR inhibitors (investigational TAK-593)	VEGFR, FGFR, CDKs	VEGFR2 kinase: 0.95 nM	Electron-deficient aromatic scaffold; suitable for kinase optimization	Kinase inhibition	Moderate; emerging scaffold for targeted radiopharmaceuticals	[67,68,69]
**Pyrimidines**	Abemaciclib	CDK4/6	CDK4 2 nM, CDK6 10 nM	Nucleobase mimic	Cell-cycle inhibition	Moderate–high; adaptable for multifunctional conjugates	[70]
**Quinazolines**	Gefitinib Afatinib Osimertinib	EGFR (ErbB family)	Gefitinib: EGFR 33 nM; Afatinib: WT EGFR 0.5 nM; Osimertinib: mutant-selective, assay- and genotype-dependent	ATP-mimetic privileged scaffold	EGFR inhibition	High target specificity; suitable for radiolabeled conjugates after chelator incorporation	[35,44,62,71,72,73]
**Metal-based scaffolds**	Cisplatin Cu-ATSM	DNA; hypoxia-associated pathways	Not directly applicable; cytotoxic potency is cell- and assay-dependent	Intrinsic coordination chemistry; variable oxidation states	DNA damage; ROS generation; metal-mediated cytotoxicity	Excellent–direct incorporation of therapeutic and diagnostic radionuclides	[37]
**Hybrid scaffolds**	PARP inhibitor-DNA damaging hybrids; TSC-heterocycle hybrids	Multiple molecular targets	Not directly applicable; compound- and target-dependent	Complementary pharmacophores within a single molecule	Modulation of multiple signaling pathways	High; enables integration of targeting motifs and radionuclides	[74]

* Radiotheranostic potential was qualitatively assessed based on the combination of target specificity, compatibility with radionuclide incorporation, structural modularity, availability of clinically relevant examples, and translational evidence reported in the literature. ALK—anaplastic lymphoma kinase; CDKs—cyclin-dependent kinases (CDKs); Cu-ATSM—copper(II)-diacetyl-bis(N4-methylthiosemicarbazone); EGFR—epidermal growth factor receptor; MET—mesenchymal–epithelial transition factor; PARP—poly(ADP-ribose) polymerase; ROS—reactive oxygen species; ROS1—c-ros oncogene 1; RR—ribonucleotide reductase; VEGFR—vascular endothelial growth factor receptor.

**Table 2:** Structural determinants of radiotheranostic suitability.

Determinant *	Definition	Impact on Translation	Ref.
**Target affinity**	Specific interaction with tumor-associated targets	Improved accumulation and imaging contrast	[35,36]
**Metal coordination**	Ability to bind diagnostic or therapeutic radionuclides	Stable radiolabeling	[37,38]
**Structural modularity**	Possibility of scaffold modification	Optimization flexibility	[55]
**Pharmacokinetic tunability**	Control of ADME properties	Improved biodistribution	[40]
**Radiosensitization potential**	Ability to enhance radiation-induced damage	Greater therapeutic efficacy	[55,65]
**Hybridization capability**	Compatibility with multifunctional design	Integrated theranostic platforms	[74]

* The proposed framework represents the authors’ synthesis of the principal structural determinants repeatedly identified across contemporary medicinal chemistry and the radiotheranostic literature and is intended as a conceptual guide rather than a quantitative scoring system. ADME—absorption, distribution, metabolism, and excretion.

## Data Availability

No new data were created or analyzed in this study. Data sharing is not applicable to this article.

## Abbreviations

The following abbreviations are used in this manuscript:

ADME	Absorption, distribution, metabolism, and excretion
AI	Artificial intelligence
CDK	Cyclin dependent kinase
Cu-ATSM	Copper(II)-diacetyl-bis(N4-methylthiosemicarbazone)
EGFR	Epidermal growth factor receptor
FAPI	Fibroblast activation protein inhibitor
HDAC	Histone deacetylase
MTDL	Multitarget-directed ligand
PARP	Poly(ADP-ribose) polymerase
PET	Positron emission tomography
PROTAC	Proteolysis-targeting chimera
PSMA	Prostate-specific membrane antigen
ROS	Reactive oxygen species
RR	Ribonucleotide reductase
SAR	Structure–activity relationship
SPECT	Single-photon emission computed tomography
TSC	Thiosemicarbazones
